# Selenium-modified bone cement promotes osteoporotic bone defect repair in ovariectomized rats by restoring GPx1-mediated mitochondrial antioxidant functions

**DOI:** 10.1093/rb/rbad011

**Published:** 2023-02-14

**Authors:** Quan Zhou, Weikai Chen, Chao Gu, Hao Liu, Xiayu Hu, Lei Deng, Wei He, Yong Xu, Xuesong Zhu, Huilin Yang, Xi Chen, Fan He, Tao Liu

**Affiliations:** Department of Orthopaedics, The First Affiliated Hospital of Soochow University, Suzhou 215006, China; Orthopaedic Institute, Medical College, Soochow University, Suzhou 215007, China; Department of Orthopaedics, The First Affiliated Hospital of Soochow University, Suzhou 215006, China; Orthopaedic Institute, Medical College, Soochow University, Suzhou 215007, China; Orthopaedic Institute, Medical College, Soochow University, Suzhou 215007, China; Department of Orthopaedics, Suzhou Dushu Lake Hospital, Suzhou 215125, China; Department of Orthopaedics, The First Affiliated Hospital of Soochow University, Suzhou 215006, China; Department of Orthopaedics, The First Affiliated Hospital of Soochow University, Suzhou 215006, China; Orthopaedic Institute, Medical College, Soochow University, Suzhou 215007, China; Department of Orthopaedics, The First Affiliated Hospital of Soochow University, Suzhou 215006, China; Orthopaedic Institute, Medical College, Soochow University, Suzhou 215007, China; Department of Orthopaedics, The First Affiliated Hospital of Soochow University, Suzhou 215006, China; Orthopaedic Institute, Medical College, Soochow University, Suzhou 215007, China; Orthopaedic Institute, Medical College, Soochow University, Suzhou 215007, China; Department of Orthopaedics, The First Affiliated Hospital of Soochow University, Suzhou 215006, China; Department of Orthopaedics, The First Affiliated Hospital of Soochow University, Suzhou 215006, China; Orthopaedic Institute, Medical College, Soochow University, Suzhou 215007, China; Department of Pathology, The Third Affiliated Hospital of Soochow University, Changzhou 213003, China; Department of Orthopaedics, The First Affiliated Hospital of Soochow University, Suzhou 215006, China; Orthopaedic Institute, Medical College, Soochow University, Suzhou 215007, China; Department of Orthopaedics, The First Affiliated Hospital of Soochow University, Suzhou 215006, China

**Keywords:** sodium selenite, osteoporosis, GPx1, mitochondrial function, bone cement

## Abstract

Over-accumulation of reactive oxygen species (ROS) causes mitochondrial dysfunction and impairs the osteogenic potential of bone marrow-derived mesenchymal stem cells (BMMSCs). Selenium (Se) protects BMMSCs from oxidative stress-induced damage; however, it is unknown whether Se supplementation can promote the repair of osteoporotic bone defects by rescuing the impaired osteogenic potential of osteoporotic BMMSCs (OP-BMMSCs). *In vitro* treatment with sodium selenite (Na_2_SeO_3_) successfully improved the osteogenic differentiation of OP-BMMSCs, as demonstrated by increased matrix mineralization and up-regulated osteogenic genes expression. More importantly, Na_2_SeO_3_ restored the impaired mitochondrial functions of OP-BMMSCs, significantly up-regulated glutathione peroxidase 1 (GPx1) expression and attenuated the intracellular ROS and mitochondrial superoxide. Silencing of *Gpx1* completely abrogated the protective effects of Na_2_SeO_3_ on mitochondrial functions of OP-BMMSCs, suggesting the important role of GPx1 in protecting OP-BMMSCs from oxidative stress. We further fabricated Se-modified bone cement based on silk fibroin and calcium phosphate cement (SF/CPC). After 8 weeks of implantation, Se-modified bone cement significantly promoted bone defect repair, evidenced by the increased new bone tissue formation and enhanced GPx1 expression in ovariectomized rats. These findings revealed that Se supplementation rescued mitochondrial functions of OP-BMMSCs through activation of the GPx1-mediated antioxidant pathway, and more importantly, supplementation with Se in SF/CPC accelerated bone regeneration in ovariectomized rats, representing a novel strategy for treating osteoporotic bone fractures or defects.

## Graphical Abstract

**Figure:**
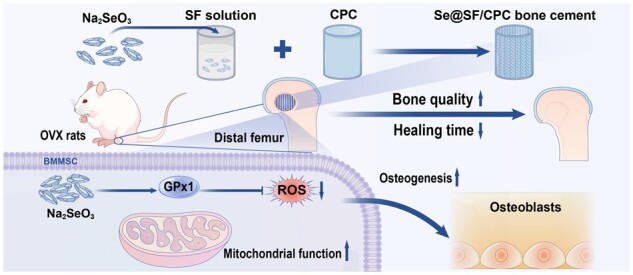

## Introduction

Osteoporosis (OP) is a progressive systemic skeletal disease characterized by the loss of bone mass and deterioration of bone microstructure [1]. A striking reduction in the callus size of bone fractures and defects can be caused by OP [2]. Worldwide, OP causes over 8.9 million bone fractures yearly and affects 200 million women [3], and in China, it is estimated that by 2035 and 2050, the number of patients with osteoporotic fractures will reach 4.83 million and 5.99 million, respectively [4]. Additionally, the healing time of bone fractures or defects has been significantly longer in patients with OP than that in healthy people [5]. The most common problems associated with the osteoporotic healing process include long healing time, poor healing ability, and a high rate of bone nonunion, so improving bone regeneration in patients with osteoporotic fractures is of great interest.

Bone marrow-derived mesenchymal stem cells (BMMSCs) are an important cell source for bone formation that can potentially differentiate into osteoblasts, chondrocytes, adipocytes etc. [6]. Many studies have demonstrated that BMMSCs play a critical role in bone healing by migrating to sites of bone fractures or defects [7]. In addition, BMMSCs provide antioxidant protection by secreting different types of growth factors and inflammatory factors, creating a regenerative microenvironment for bone tissue repair. However, when patients are suffering from long-term OP, reactive oxygen species (ROS) in the body significantly increase and thus lead to intracellular oxidative stress (OS) in BMMSCs [8]. Mitochondria are the prime source of intracellular ROS, and excessive levels can damage mitochondrial functions, including injuring the respiratory chain of mitochondrial inner membrane, changing the permeability of mitochondrial membrane, and hindering the process of oxidative phosphorylation [9, 10]. This mitochondrial dysfunction, in turn, may disturb the redox balance of BMMSCs and inhibit their proliferation and differentiation into osteoblasts, reducing the formation of bone trabeculae and ultimately resulting in the failure of bone regeneration.

Selenium (Se), an essential trace mineral element closely related to bone health [11], plays an important role in regulating stem cell proliferation and differentiation [12]. Se-deficient mice demonstrated lower femur trabecular bone volume to total volume ratio (BV/TV) but greater bone trabecular spacing compared with normal mice, and Se deficiency is responsible for the impairment of bone microstructure by increasing bone absorption through over-accumulation of ROS [13]. Se exists primarily in the form of selenoproteins and serves the antioxidant function through various selenoproteins [14]. The most abundant selenoproteins in mammals are Se-dependent glutathione peroxidases (GPx); they are important antioxidant enzymes with protection and detoxification effects, in which Se has been identified as part of the active center. In addition, GPx regulates and stores Se, which can be broken down and released when the body needs it [15]. Among these selenoproteins, glutathione peroxidase 1 (GPx1) is a critical antioxidant enzyme that is distributed in the cytoplasm and mitochondria [16]. Sodium selenite (Na_2_SeO_3_) has been used as an oral Se supplement, whose effect has been clinically confirmed. In previous studies, Na_2_SeO_3_ was reported to improve the antioxidant capacity of stem cells at safe doses [17, 18]. However, it is still unclear whether Na_2_SeO_3_ could improve the bone formation ability of osteoporotic BMMSCs (OP-BMMSCs), and the underlying mechanisms involved in GPx1-mediated antioxidant function require further investigation.

Recently, calcium phosphate cement (CPC) has been widely recognized as one of the most suitable injectable artificial biomaterials to treat narrow and irregular bone defects [19]. In addition, recent studies have confirmed that some inorganic metal ions or drug molecules combined with CPC can promote the repair of osteoporotic fractures or bone defects [20, 21]. Silk fibroin (SF) is a kind of natural high molecular protein secreted by silkworm and has a wide array of medical applications owing to its good biocompatibility, degradability, modification and stable mechanical properties [22, 23]. It has been reported that SF combined with CPC can be used for implant fixation and bone tissue defects [24], and both CPC and SF have good loading properties for various drugs and biological factors [25, 26]. However, the therapeutic effects of SF/CPC scaffold on osteoporotic bone defects are unsatisfactory due to the limited bone inducibility. Therefore, we prepared a Se-modified bioactive SF/CPC scaffold and hypothesized that Se-modified bone cement could improve osteoporotic bone defect repair in ovariectomized (OVX) rats. In this study, the effects of Na_2_SeO_3_ on osteogenic differentiation potential and mitochondrial antioxidant functions of OP-BMMSCs were explored, and the underlying role of GPx1 was also investigated.

## Materials and methods

### Reagents and antibodies

Fetal bovine serum (FBS), alpha minimum essential medium (αMEM), penicillin, streptomycin, TRIzol^®^ reagent, Dulbecco’s modified Eagle medium (DMEM), RevertAid First Strand cDNA Synthesis Kit, chemiluminescence solution, 4′,6-diamidino-2-phe-nylindole (DAPI), and trypsin–ethylene diamine tetraacetic acid (EDTA) were purchased from Thermo Fisher Scientific (Waltham, MA, USA). Na_2_SeO_3_, ethanol (EtOH), paraformaldehyde, l-ascorbic acid, dexamethasone, β-glycerol phosphate, phosphate-buffered saline (PBS), alizarin red S (ARS), pentobarbital and calcium hydrogen phosphate dihydrate (CaHPO_4_·2H_2_O, DCPD) were obtained from Sigma–Aldrich (St. Louis, MO, USA). Red blood cell lysis buffer, counting kit-8 (CCK-8), 5% perchloric acid solution, cell lysis buffer, bicinchoninic acid (BCA) protein assay kit, adenosine 5′-triphosphate (ATP) assay kit, 5,5ʹ,6,6ʹ-tetrachloro-1,1ʹ,3,3ʹ-tetraethylbenzimidazolyl carbo-cyanine iodide (JC-1) dye, 2′,7′-dichlorofluorescein diacetate (DCFH-DA) and nitrocellulose membrane were purchased from Beyotime (Haimen, China). Primary antibodies against CD29 (ab36219), CD90 (ab25672), CD34 (ab23830), CD45 (ab210225), GPx1 (ab22604), ATP5A (ab176569), ND4 (ab219822), SDHA (ab137040), COX4 (ab202554), β-actin (ab8227) and goat anti-rabbit IgG (H&L) (ab150079) were purchased from Abcam (Cambridge, MA, USA).

### Establishment of OVX rat models

Sprague–Dawley rats (10-week-old, female) were purchased from the Animal Center of Soochow University. After anesthesia with pentobarbital (3%, 40 mg/kg body weight, intraperitoneally), all rats underwent either sham surgery or bilateral OVX surgery. Each rat was intramuscularly injected with a total of 6000 units of penicillin immediately after surgery.

### *In vitro* culture and differentiation of BMMSCs

#### Isolation and identification of BMMSCs

BMMSCs were isolated from bilateral femurs and tibias immediately after euthanasia of rats fed for 3 months. BMMSCs in the bone marrow cavity were flushed with αMEM, and then red blood cells were removed with red blood cell lysis buffer. BMMSCs were placed in 75-cm^2^ culture flasks containing 10 ml standard medium (αMEM supplemented with 10% FBS, 100 U/ml penicillin and 100 μg/ml streptomycin) at 37°C with 5% CO_2_. All cells used in the subsequent experiments were of passage 2.

The cultured cells were dissociated with 0.25% trypsin–EDTA. After washing with PBS, cells were incubated with monoclonal antibodies against CD29, CD90, CD34 and CD45 on ice for 30 min in the dark. Then the cell suspension (1 × 10^5^ cells) was detected by a Guava Easy Cell Flow Cytometer (FCM, Millipore, Boston, MA, USA) and analyzed by the FlowJo software (TreeStar, San Carlos, CA, USA).

#### Cell proliferation assay

BMMSCs were inoculated into 96-well plates (2 × 10^3^ per well). After cell culture to the target time, 10 μl of CCK-8 solution was added to each well for 1 h and incubated at 37°C with 5% CO_2_. Absorbance was measured at 450 nm using a PowerWave XS spectrophotometer (BioTek, Winooski, VT, USA).

#### Osteogenic differentiation and ARS staining

After BMMSCs were cultured in osteogenic medium (DMEM containing 10% FBS, 10 mM β-glycerol phosphate, 100 nM dexamethasone, 50 μg/ml l-ascorbic acid, 100 U/ml penicillin and 100 μg/ml streptomycin) for 21 days, and they were fixed in 4% paraformaldehyde (pH = 7.4) for 30 min and then incubated in 0.1% ARS solution (pH = 4.2) at room temperature for 15 min. Calcium salt deposition was observed via an inverted microscope (Olympus, Japan). For quantitative analysis, 5% perchloric acid solution was added to dissolve the stain from the calcified layer. The absorbance was measured at 420 nm using a PowerWave XS spectrophotometer.

### Total RNA extraction and quantitative real-time reverse transcription–polymerase chain reaction

Total RNA from BMMSCs was extracted by adding TRIzol^®^ reagent, and complementary DNA (cDNA) was synthesized using the RevertAid First Strand cDNA Synthesis Kit. Subsequently, real-time reverse transcription–polymerase chain reaction (RT-PCR) was performed using the iTap™ Universal SYBR^®^ Green Supermix kit (Bio-Rad, Hercules, CA, USA) on a CFX96™ Real-Time RT-PCR System (Bio-Rad). Relative transcription levels were calculated by applying the 2^−ΔΔCt^ method, and these levels were normalized to the *Gapdh* transcription level. Primer sequences are enumerated in Supplementary Table S1.

### Western blotting

Total protein of BMMSCs were extracted using cell lysis buffer, and the BCA protein assay kit was used to determine the concentration. An equal amount of protein from each extract was denatured at 100°C for 5 min, separated by electrophoresis in a 10% polyacrylamide gel, and transferred onto a nitrocellulose membrane. Membranes were blocked for 30 min and incubated with properly diluted primary antibodies overnight at 4°C. The membranes were incubated in secondary antibodies (1:2000, Abcam) conjugated to horseradish peroxidase at room temperature for 1 h. The membranes were incubated in chemiluminescence solution to enhance visualization of the proteins, and ImageJ software (National Institutes of Health, Bethesda, MD, USA) was used to assess the band intensity.

### Measurement of GPx enzyme activity

The methods of protein extraction and concentration determination were described by Western blotting assay. The enzyme activity of total GPx was measured using a commercial test kit (Beyotime, S0058) following the manufacturer’s instructions. The decrease in nicotinamide adenine dinucleotide phosphate measured at 340 nm was linearly correlated with enzyme activity using a PowerWave XS spectrophotometer.

### Immunofluorescence staining

BMMSCs in 24-well plates were fixed in 4% paraformaldehyde for 15 min and permeabilized by 0.1% Triton X-100 (Sigma-Aldrich) for 10 min. The cells were blocked in 1% bovine serum for 30 min and incubated for 1 h in anti-GPx1 primary antibody (1:200). After washing with PBS, the cells were incubated with goat anti-rabbit IgG (H&L) for 1 h. The cytoskeleton F-actin was stained with Phalloidin-iFluor 594 Reagent (Abcam) for 20 min and the nucleus was counterstained using DAPI. Immunofluorescence images were captured using a Zeiss Axiovert 40CFL microscope (Zeiss, Oberkochen, Germany).

### Mitochondrial function and antioxidant function assay

#### Intracellular ROS measurement

BMMSCs were dissociated with 0.25% trypsin–EDTA, after which the cell suspension (1 × 10^6^ cells) was incubated in 10 μM DCFH-DA solution at 37°C for 10 min. Fluorescence intensity was measured at a wavelength (excitation/emission) of 488/530 nm using a Guava Easy Cell Flow Cytometer. The ROS level was analyzed using FlowJo software. For immunofluorescence staining, cells were incubated with 10 μM DCFH-DA solution for 10 min at 37°C in the dark, and the nucleus was counterstained using DAPI. The fluorescence images were captured using a Zeiss fluorescence microscope, and the fluorescence intensity was measured using the ImageJ software.

#### Measurement of mitochondrial superoxide

The mitochondrial superoxide level was measured using a mitochondria-specific indicator MitoSOX™ Red (Molecular Probes™, Invitrogen). BMMSCs were loaded with 5 μM MitoSOX™ Red in the dark for 10 min at 37°C. The fluorescence intensity of cells (10 000) in each group was measured using a Guava EasyCyte Flow Cytometer and analyzed using FlowJo software.

#### Mitochondrial membrane potential MMP measurement

Dissociated BMMSCs were incubated with JC-1 dye at 37°C in darkness for 20 min. The fluorescence intensity at an excitation wavelength of 510 nm and an emission wavelength of 527 nm was recorded by a Guava EasyCyte Flow Cytometer.

#### Mitochondrial ATP concentration

With 20 µl of the supernatant, 100 µl of ATP detection working solution was added to a black 96-well plate for luminescence analysis using a Centro LB 960 (Berthold Technologies, Germany). The protein concentration in each well was measured using a BCA protein assay kit to normalize the relative ATP concentration.

### Small interfering RNA transfection

The small interfering RNA (siRNA) targeting *Gpx1* (siGPx1) was mixed with Lipofectamine™ 2000 (Invitrogen Life Technologies) diluent and incubated at room temperature for 20 min. After treating with siRNA and Lipofectamine™ 2000 mixture, BMMSCs were cultured in an incubator for 6 h at 37°C with 5% CO_2_. Finally, the cells were washed with PBS, and a fresh culture medium was added for subsequent experiments.

### Modified bone cement preparation

The ratio of materials required to prepare cement powder was as follows: 87% α-tricalcium phosphate (α-TCP) purchased from Ensail Co., Ltd (Beijing, China) & 10% DCPD & 3% hydroxyapatite (HA). The reactive liquids added to the bone cement powder were 5% SF solution with or without the 100 μM concentration of Na_2_SeO_3_. The solid content was mixed with the corresponding reactive liquids to prepare the modified bone cement paste at a liquid–solid ratio of 0.5 ml/g.

### Characterization of modified bone cement

#### Injectability

The modified bone cement was loaded into a standard syringe with a capacity of 1 ml and an inner diameter of 2 mm and was slowly extruded at a speed of 10 mm/min until the force reached 150 N. The injectability, *I*, was calculated by the following formula: *I* = [(*M*_0_ − *M*_1_)/*M*_0_] × 100%, where *M*_0_ is the initial mass, and *M*_1_ is the remaining mass in the syringe.

#### Setting time

The time when the light needle (113.4 g, diameter 2.12 mm) could not be inserted into the top surface of the modified bone cement 1.5 mm deep was considered the initial setting time, whereas the time when the heavy needle (453.6 g, diameter 1.06 mm) could not leave a complete round dent on the surface of the modified bone cement was considered the final setting time.

#### Compressive strength

The modified bone cement in the cylindrical mold (diameter 6 mm, height 12 mm) was compressed at a cross-head speed of 1 mm/min on a universal mechanical tester (HY-1080, HengYi Precision Instrument Co., Ltd, Shanghai, China). When the modified bone cement was destroyed, the compressive strength was calculated from the recorded maximum stress value.

#### Degradability

The modified bone cement set in the cylindrical mold was immersed in PBS solution at 37°C. After drying at 60°C for 6 h, the weight loss, *W*, was calculated by using the following formula: *W* = [(*W*_0_ − *W_x_*)/*W*_0_] × 100%, where *W*_0_ was the weight when modified bone cement was not immersed in the solution, and *W_x_* was the weight each time after it was taken out of the solution and then dried.

#### Scanning electron microscope and energy dispersive X-ray spectroscopy analysis

The fresh fractured surfaces of modified bone cement samples were examined by scanning electron microscope (SEM) (Regulus SU8100, Japan) on the gold-coated samples at an acceleration voltage of 2 kV. The atomic compositions of modified bone cement were measured using SEM equipped with X-ray spectroscopy (EDS), and the acceleration voltage was 20 kV.

#### X-ray diffraction measurement

X-ray powder diffraction (XRD) was performed using an X-ray diffractometer (Bruker D8 Advance, Germany) at 40 kV and 40 mA to monitor the crystalline nature of the Se-modified bone cement. Data were analyzed from a 2θ value between 5° and 85° under CuKα radiation.

#### In vitro Na_2_SeO_3_ release assay

The concentration of Na_2_SeO_3_ released by modified bone cement was determined by UV–vis spectrophotometer (Shimadzu Co., LTD, Shanghai, China). The completely solidified modified bone cement was immersed in PBS and rotated in a 37°C mixer (120 RPM) for 20 days. According to the standard curve of Na_2_SeO_3_, the absorbance value at 205 nm was read to calculate the concentration of Na_2_SeO_3_ released from the modified bone cement.

#### Biocompatibility

Sterilized modified bone cement was added to the PBS at 0.2 g/ml and left for 24 h to extract the leach solution. One milliliter of the leach solution was added into 99 ml cell culture medium for the subsequent experiments. A total of 1 × 10^4^ cells per well were cultured with the leach solution, and the culture medium without the leach solution was used as the CTRL group. In the cell proliferation assay, cells were treated with 10% CCK-8 solution and observed using a 450 nm wavelength microplate reader. To evaluate the cytotoxicity, cells were incubated with live/dead reagent (Termo Fisher Scientific) at a temperature of 37°C for 15 min. The live (green) or dead (red) cells were captured using a Zeiss fluorescence microscope.

### *In vivo* experiments

#### Establishment of bone defect model in OVX rats

According to the rat model described in the animal experiment above, all normal and OVX rats were randomly assigned to the sham group, the SF/CPC group or the Se@SF/CPC group, with 10 rats in each group. After the rats were anesthetized with pentobarbital, a diameter of 3 mm and a depth of 5 mm at the distal lateral epididymis of each femur were drilled. Subsequently, the tunnel of the femoral defect was filled with the prepared modified bone cement in the SF/CPC and the Se@SF/CPC groups. All rats were given 6000 units of penicillin intramuscular injection immediately after surgery.

#### Micro-CT scanning and analysis

The microstructural feature of the bone defect was analyzed by using a high-resolution μCT system (Skyscan 1176, Kontich, Belgium). The site of the bone defect in the distal femur as the region of interest (ROI) was identified, and the scanning resolution of the ROI was 18 μm, and the scanning energy was 65 kV and 385 μA. The NRecon v1.6 and CTAn v1.13.8.1 software were used for 3D reconstruction. BV/TV was calculated to analyze the healing process of the bone microstructure in the ROI.

#### Histological and immunohistochemical analysis

After decalcification using 10% EDTA, bone specimens were dehydrated in graded EtOH solutions and embedded in paraffin. Paraffin-embedded tissue samples were sectioned sagittally at a thickness of 5 μm. For histological analysis, the bone structures of each sample were observed by staining with hematoxylin eosin (H&E) and Masson (Solaibao Technology Co., Ltd, Beijing, China) following each manufacturer’s protocol. Histological images were obtained using a bright field microscope. For immunohistochemical staining, slides were incubated with anti-GPx1 primary antibody overnight at 4°C. The sections were then incubated with a biotinylated secondary antibody and stained with 3,3′-diaminobenzidine solution (DAB, Vector Laboratories, Burlingame, CA, USA). The percentage of GPx1-positive cells was quantified using Image J software.

### Statistical analysis

All data were expressed as the mean ± standard error of the mean (SEM). The two-tailed Student’s *t*-test was used to compare the two groups, and one-way analysis of variance (ANOVA) was implemented for comparisons among multiple groups. Statistically significant differences were indicated by *P <* *0.05 or **P <* *0.01 between the indicated groups. All statistical analyses were performed using SPSS 18.0 statistical software (SPSS Inc., Chicago, IL, USA).

## Results

### OP-BMMSCs show decreased osteogenic potential and impaired mitochondrial antioxidant functions

**Figure 1.**
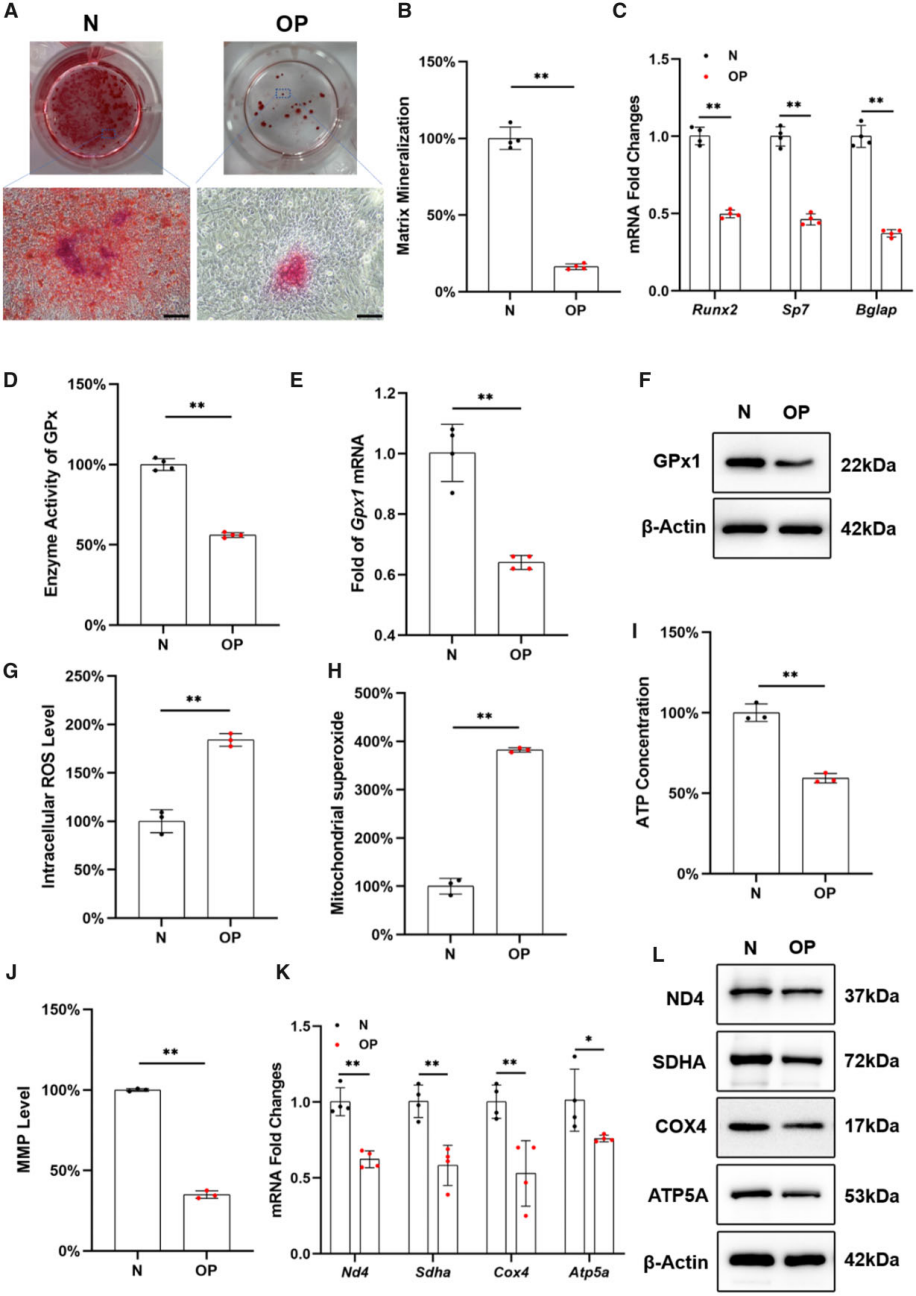
BMMSCs derived from OVX rats (OP-BMMSCs) exhibited decreased osteogenic potential, increased OS and impaired mitochondrial functions. (**A**) BMMSCs derived from normal (N-BMMSCs) and OVX rats (OP-BMMSCs) were induced toward osteogenic differentiation for 21 days. Representative images of mineralized extracellular matrix stained by ARS. Scale bar = 200 μm. (**B**) Comparison of matrix mineralization between N-BMMSCs and OP-BMMSCs, *n* = 4. (**C**) The gene expressions of osteoblast-specific markers were quantified with real-time RT-PCR, *n* = 4. (**D**) The enzyme activity of total GPx was compared between N-BMMSCs and OP-BMMSCs, *n* = 4. (**E**) The transcript level of *Gpx1* was quantified with real-time RT-PCR, *n* = 4. (**F**) The protein level of GPx1 protein was determined using Western blot, *n* = 3. (**G**) The intracellular ROS was measured by flow cytometry, *n* = 3. (**H**) The mitochondrial superoxide level was compared between N-BMMSCs and OP-BMMSCs, *n* = 3. (**I**) ATP content was evaluated, *n* = 3. (**J**) MMP level was determined by flow cytometry, *n* = 3. (**K**) The gene expressions of *Nd4, sdha, Cox4* and *Atp5a* were quantified with real-time RT-PCR, *n* = 4. (**L**) The protein levels of ND4, SDHA, COX4 and ATP5A were determined using Western blot, *n* = 3. Values represent mean ± SEM. Statistically significant differences are indicated by * where *P *<* *0.05 or ** where *P *<* *0.01 between the indicated groups.

BMMSCs from osteoporotic rats showed strong expression of CD29 and CD90 markers, while negative for CD34 or CD45, suggesting the similar cell surface expression to that of MSCs (Supplementary Fig. S1A). The proliferation of OP-BMMSCs was significantly lower than that of normal BMMSCs (N-BMMSCs) (Supplementary Fig. S1B). After a 21-day osteogenic induction, the mineral deposition level of OP-BMMSCs was 83.7% lower than that of N-BMMSCs (Fig. 1A and B), and the transcription levels of osteogenic markers in OP-BMMSCs, including *Runx2*, *Sp7* and *Bglap*, were significantly down-regulated (Fig. 1C). Compared with N-BMMSCs, the enzyme activity of total GPx in OP-BMMSCs was significantly decreased by 43.9% (Fig. 1D), and the transcription and protein levels of GPx1 were significantly down-regulated by 36.0% and 32.2%, respectively (Fig. 1E and F; Supplementary Fig. S1C). In addition, intracellular ROS and mitochondrial superoxide levels of OP-BMMSCs were 1.8- and 3.8-fold higher than those of N-BMMSCs, respectively (Fig. 1G and H). Compared with N-BMMSCs, ATP concentration and Mitochondrial membrane potential (MMP) level of OP-BMMSCs were significantly reduced by 40.7% and 65.1%, respectively (Fig. 1I and J; Supplementary Fig. S1D). Concerning mitochondrial respiratory chain function, both the transcription and protein levels of mitochondrial respiratory factors of OP-BMMSCs, including SDHA, COX4, ATP5A and ND4, were significantly down-regulated (Fig. 1K and L; Supplementary Fig. S1E).

### *In vitro* supplementation with Na_2_SeO_3_ rescued the osteogenic differentiation of OP-BMMSCs

**Figure 2.**
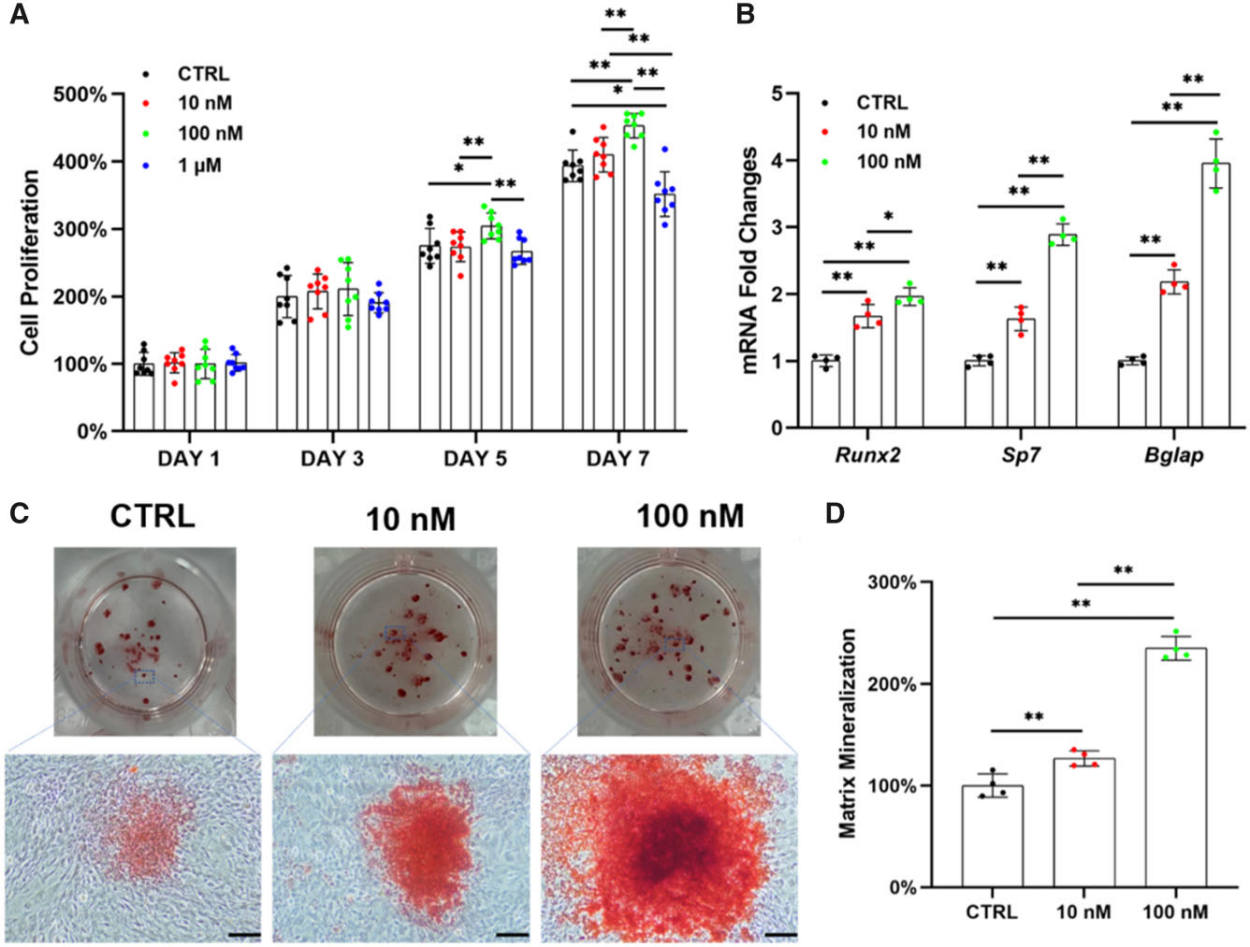
*In vitro* supplementation with Na_2_SeO_3_ rescued the proliferation and osteogenic differentiation of OP-BMMSCs. The cells were treated with 10, 100 nM and 1 μM of Na_2_SeO_3_, while untreated cells served as the control (CTRL) group. (**A**) Proliferation of Na_2_SeO_3_-treated OP-BMMSCs was examined on Days 1, 3, 5 and 7 using CCK-8 assay, *n* = 8. (**B**) The gene expressions of osteoblast-specific markers in Na_2_SeO_3_-treated OP-BMMSCs were quantified with real-time RT-PCR, *n* = 4. (**C**) Representative images of mineralized extracellular matrix stained by ARS. Scale bar = 200 μm, *n* = 4. (**D**) Quantification of the stained mineral layers of Na_2_SeO_3_-treated OP-BMMSCs, *n* = 4. Values represent mean ± SEM. Statistically significant differences are indicated by * where *P *<* *0.05 or ** where *P *<* *0.01 between the indicated groups.

To investigate the effects of Na_2_SeO_3_ on OP-BMMSCs *in vitro*, the cells were treated with 10, 100 nM and 1 μM of Na_2_SeO_3_. The proliferation of OP-BMMSCs was significantly improved by treatment with 100 nM of Na_2_SeO_3_, while it was significantly inhibited at the concentration of 1 μM (Fig. 2A). Real-time RT-PCR revealed that the transcription levels of osteogenic markers were significantly up-regulated with the treatment of Na_2_SeO_3_ (Fig. 2B). After a 21-day osteogenic induction, treatments with 10 and 100 nM of Na_2_SeO_3_ significantly increased the matrix mineralization of OP-BMMSCs that were 1.3- and 2.4-fold higher than that of the control (CTRL) group, respectively (Fig. 2C and D).

### *In vitro* Na_2_SeO_3_ restored the mitochondrial antioxidant functions of OP-BMMSCs

**Figure 3.**
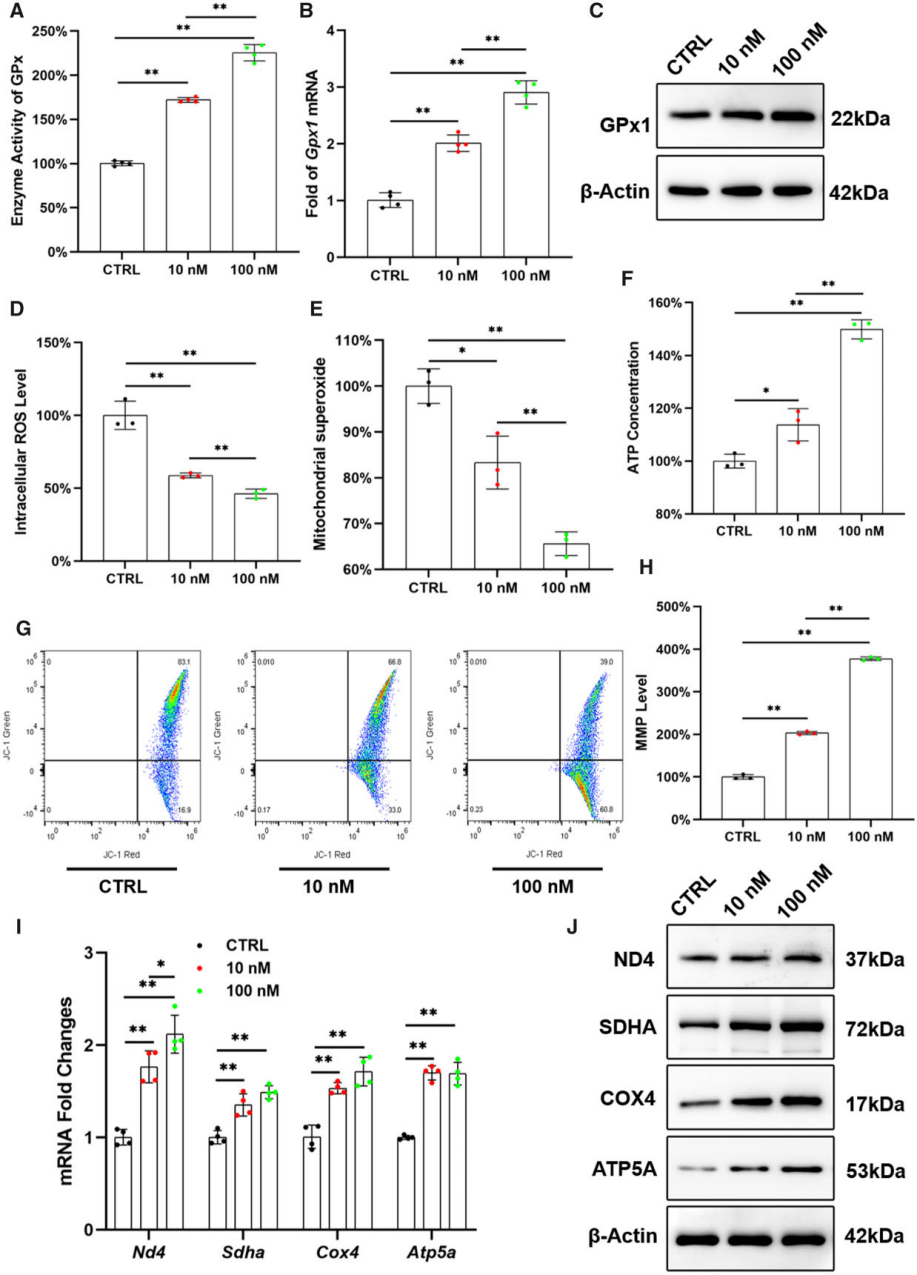
*In vitro* treatment with Na_2_SeO_3_ attenuated intracellular OS and restored the mitochondrial functions of OP-BMMSCs. (**A**) Na_2_SeO_3_ increased the enzyme activity of total GPx in a dose-dependent manner, *n* = 4. (**B**) *Gpx1* gene expression was quantified with real-time RT-PCR, *n* = 4. (**C**) The protein level of GPx1 was determined using Western blot, *n* = 3. (**D**) Treatment with Na_2_SeO_3_ significantly reduced the intracellular ROS, *n* = 3. (**E**) Treatment with Na_2_SeO_3_ attenuated mitochondrial superoxide level, *n* = 3. (**F**) ATP content was measured in Na_2_SeO_3_-treated OP-BMMSCs, *n* = 3. (**G**, **H**) Treatment with Na_2_SeO_3_ improved MMP in OP-BMMSCs, *n* = 3. (**I**) The gene expressions of *Nd4, sdha, Cox4* and *Atp5a* were quantified with real-time RT-PCR, *n* = 4. (**J**) The protein levels of ND4, SDHA, COX4 and ATP5A were determined using Western blot, *n* = 3. Values represent mean ± SEM. Statistically significant differences are indicated by * where *P *<* *0.05 or ** where *P *<* *0.01 between the indicated groups.

After treating with Na_2_SeO_3_*in vitro*, the GPx enzyme activity of OP-BMMSCs was significantly increased by 1.7-fold at 10 nM and 2.3-fold at 100 nM compared with the CTRL group (Fig. 3A). In addition, the transcription level of *Gpx1* was significantly increased by 2.0-fold in the 10 nM group and 2.9-fold in the 100 nM group (Fig. 3B). Western blot confirmed that treatment with Na_2_SeO_3_ could significantly enhance the protein expression of GPx1 in OP-BMMSCs (Fig. 3C; Supplementary Fig. S2A).

Flow cytometry analysis demonstrated that Na_2_SeO_3_ at 10 and 100 nM significantly attenuated OS in OP-BMMSCs, evidenced by the fact that intracellular ROS levels were significantly reduced by 41.3% and 53.8%, respectively, compared with the CTRL group (Fig. 3D). Meanwhile, mitochondrial superoxide levels were also down-regulated by the treatment with Na_2_SeO_3_ (Fig. 3E). After treating with 100 nM of Na_2_SeO_3_, the ATP content and MMP level were increased by 49.9% (Fig. 3F) and 2.8-fold (Fig. 3G and H), respectively. Both the transcription and protein levels of mitochondrial respiratory chain-related complexes in OP-BMMSCs were significantly up-regulated by treatment with Na_2_SeO_3_ (Fig. 3I and J; Supplementary Fig. S2B).

### Inhibition of *Gpx1* expression abrogated the antioxidant and osteogenic effects of Na_2_SeO_3_ on OP-BMMSCs

**Figure 4.**
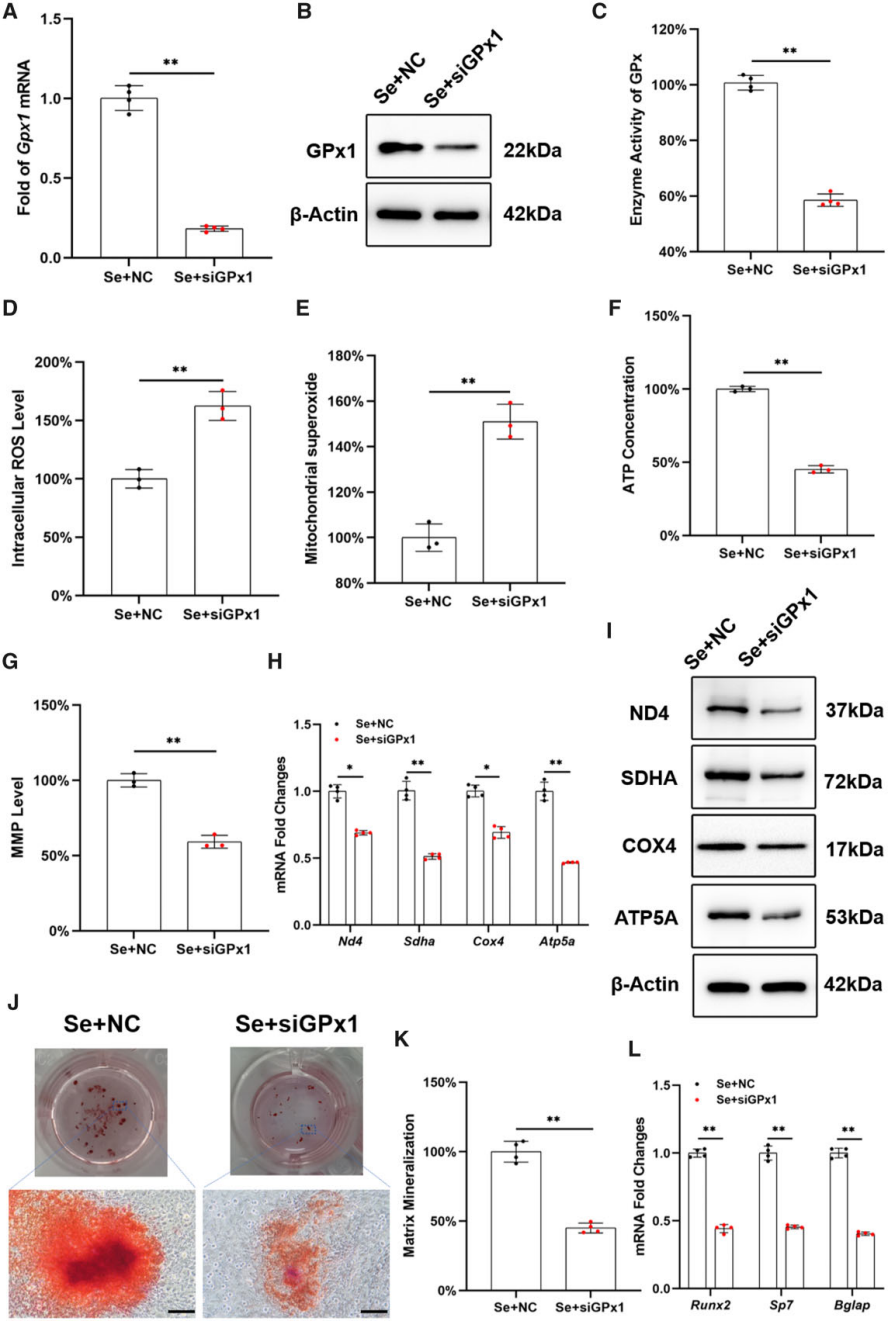
Inhibition of *Gpx1* expression by siRNA abrogated the protective effect of Na_2_SeO_3_ on the osteogenic potential and mitochondrial antioxidant functions of OP-BMMSCs. OP-BMMSCs were pre-treated with siRNA to inhibit *Gpx1* expression and then exposed to 100 nM Na_2_SeO_3_. (**A**) RT-PCR revealed that transfection with *Gpx1*-siRNA inhibited the gene expression of *Gpx1*, *n* = 4. (**B**) Western blot confirmed that the protein level of GPx1 in siRNA-treated OP-BMMSCs was significantly down-regulated, *n* = 3. (**C**) The enzyme activity of total GPx was significantly decreased after siRNA transfection, *n* = 4. (**D**) The intracellular ROS has increased in siRNA-transfected cells even exposure to Na_2_SeO_3_, *n* = 3. (**E**) Inhibition of *Gpx1* increased mitochondrial superoxide production, *n* = 3. (**F**) Transfection with *Gpx1*-siRNA reduced the ATP content in Na_2_SeO_3_-treated cells, *n* = 3. (**G**) Transfection with *Gpx1*-siRNA decreased the MMP in Na_2_SeO_3_-treated cells, *n* = 3. (**H**) Inhibition of *Gpx1* significantly down-regulated the gene expressions of *Nd4*, *sdha*, *Cox4* and *Atp5a*, *n* = 4. (**I**) Western blot confirmed that treatment with *Gpx1*-siRNA decreased the protein levels of ND4, SDHA, COX4 and ATP5A, *n* = 3. (**J**) Inhibition of *Gpx1* suppressed the matrix mineralization of Na_2_SeO_3_-treated cells. Scale bar = 200 μm, *n* = 4. (**K**) Quantification of the stained mineral layers, *n* = 4. (**L**) Inhibition of *Gpx1* down-regulated the gene expressions of osteoblast-specific markers in Na_2_SeO_3_-treated cells, *n* = 4. Values represent mean ± SEM. Statistically significant differences are indicated by * where *P *<* *0.05 or ** where *P *<* *0.01 between the indicated groups.

After transfected with siRNA, the transcription and protein levels of GPx1 were down-regulated by 81.8% and 72.6%, respectively (Fig. 4A and B; Supplementary Fig. S3A). The GPx enzyme activity was also reduced by 41.5% (Fig. 4C). Subsequently, intracellular ROS and mitochondrial superoxide levels were increased by 1.6- and 1.5-fold, respectively, in siRNA-transfected cells (Fig. 4D and E). The ATP content and MMP level were significantly reduced by 54.8% and 40.8%, respectively, by the inhibition of *Gpx1* expression (Fig. 4F and G; Supplementary Fig. S3B). Consistently, transcription and protein levels of mitochondrial respiratory chain-related factors were significantly down-regulated in siRNA-treated OP-BMMSCs (Fig. 4H and I; Supplementary Fig. S3C). Furthermore, the proliferation of Na_2_SeO_3_-treated OP-BMMSCs was significantly decreased after silencing *Gpx1* expression (Supplementary Fig. S3D). *In vitro* osteogenesis revealed that inhibition of *Gpx1* significantly suppressed matrix mineralization, which dropped by 55.0% (Fig. 4J and K). Real-time RT-PCR confirmed that gene expressions of osteogenic markers were significantly down-regulated after silencing *Gpx1* expression (Fig. 4L).

### Characterization of modified bone cement

**Figure 5.**
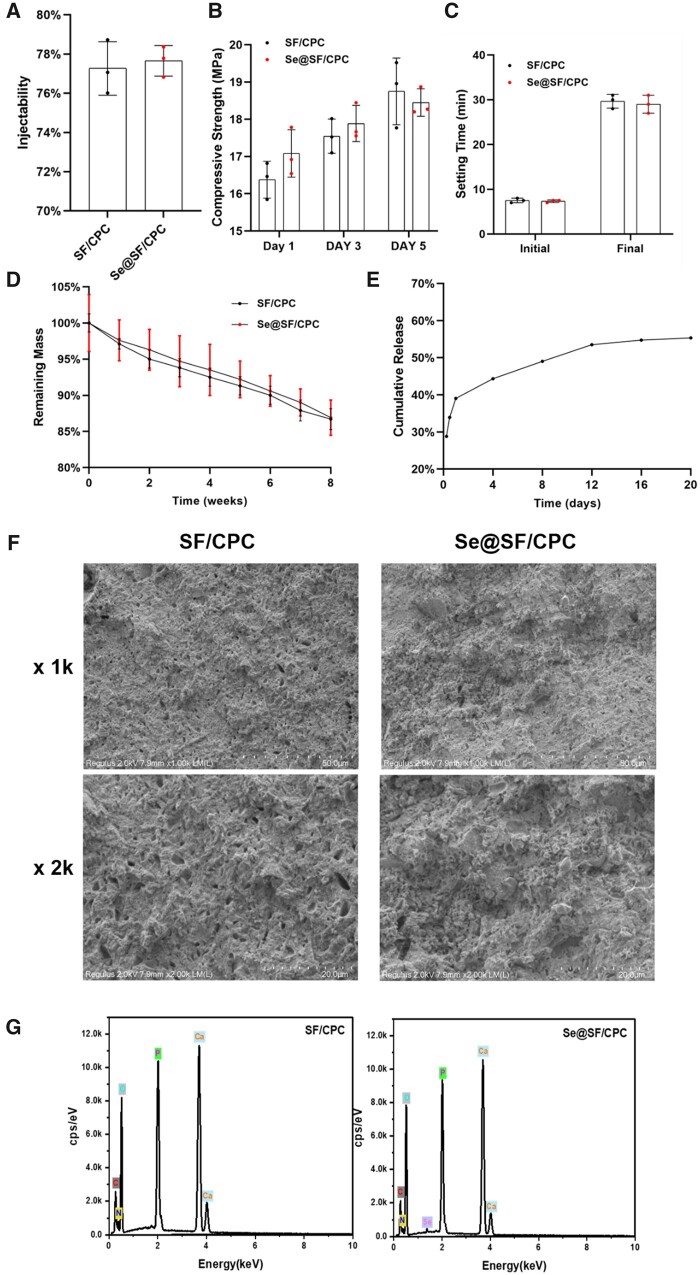
Characterization of Se-modified bone cement. Bone cement was prepared by mixing liquid silk fibroin (SF) that contained Na_2_SeO_3_ with CPC powder (Se@SF/CPC). (**A**) The effect of Na_2_SeO_3_ supplementation on the injectability of SF/CPC, *n* = 3. (**B**) The effect of Na_2_SeO_3_ supplementation on the compressive strength of SF/CPC, *n* = 3. (**C**) The initial and final setting time of SF/CPC and Se@SF/CPC were compared, *n* = 3. (**D**) The effect of Na_2_SeO_3_ supplementation on SF/CPC degradation was compared, *n* = 3. (**E**) Cumulative release of Na_2_SeO_3_ in Se@SF/CPC was determined. (**F**) Representative images of the surface morphology using an SEM. (**D**) The energy dispersive EDS elemental analysis maps of all elements, including oxygen (O), calcium (Ca), carbon (C), phosphorus (P), nitrogen (N) and selenium (Se). Values represent mean ± SEM. Statistically significant differences are indicated by * where *P *<* *0.05 or ** where *P *<* *0.01 between the indicated groups.

The injectability (Fig. 5A), compressive strength (Fig. 5B) and setting time (Fig. 5C) of bone cement were barely affected by the addition of Na_2_SeO_3_, since there was an insignificant difference between the two groups. At Week 8, the remaining mass of SF/CPC and Se@SF/CPC was 86.7% ± 1.4% and 86.9% ± 2.4%, respectively, indicating that supplementation with Na_2_SeO_3_ did not affect the degradation property of the bone cement (Fig. 5D). As demonstrated in the cumulative release assay, 53.5% of the Na_2_SeO_3_ was continuously released from the SF/CPC bone cement by Day 20 (Fig. 5E). SEM showed that there was no significant difference in the surface morphology between the two bone cements (Fig. 5F). EDS results showed there were significant peaks of oxygen (O), calcium (Ca) and phosphorus (P) in the two bone cements, and the Se peaks in Se@SF/CPC (Fig. 5G; Supplementary Fig. S4C). The XRD analysis indicated that Na_2_SeO_3_’s crystalline structure mainly peaked at a 2θ = 37.7°, and 22.1°, but Se@SF/CPC yielded an amorphous character without the formation of crystalline peaks. The results confirmed that there was a stable inclusion complex in the bone cement and Na_2_SeO_3_ supplementation had no significant effect on the XRD patterns of the SF/CPC cement (Supplementary Fig. S4B).

### Biocompatibility and antioxidant properties of modified bone cement

**Figure 6.**
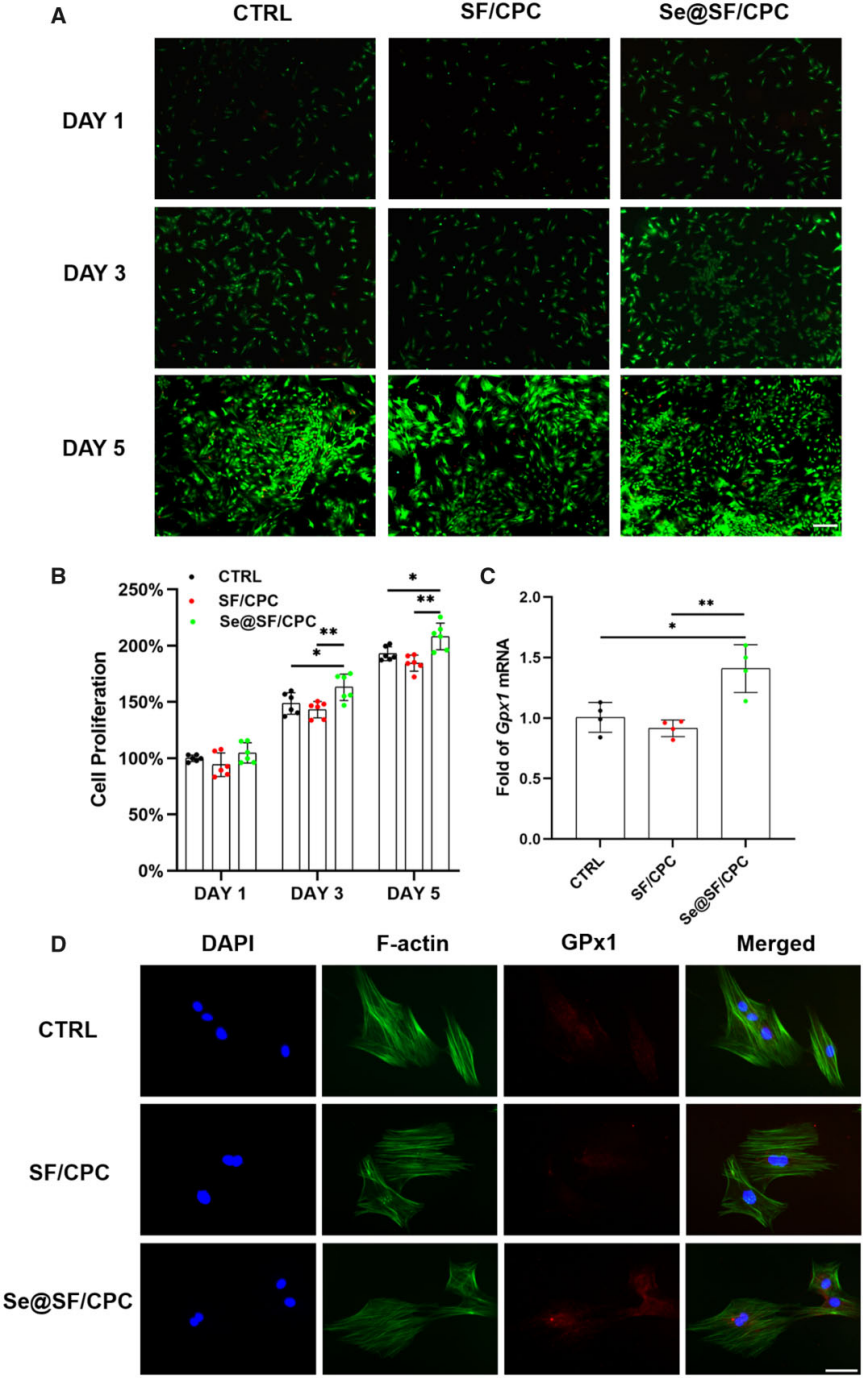
Biocompatibility of modified bone cement and the GPx1 expression. OP-BMMSCs were cultured using the leached solution of SF/CPC and Se@SF/CPC, while cell culture medium without served as the CTRL group. (**A**) Cell viability was compared by a live/death staining assay. Scale bar = 500 μm. (**B**) Cell proliferation was evaluated by CCK-8 assay between the groups of the SF/CPC, Se@SF/CPC and CTRL, *n* = 6. (**C**) The *Gpx1* gene expression of OP-BMMSCs was quantified with real-time RT-PCR, *n* = 4. (**D**) The GPx1 protein expression of OP-BMMSCs were detected by immunofluorescence assay. Scale bar: 100 μm. Values represent mean ± SEM. Statistically significant differences are indicated by * where *P *<* *0.05 or ** where *P *<* *0.01 between the indicated groups.

The living cells treated with the leaching solution of Se@SF/CPC proliferated to fusion with few dead cells (Fig. 6A), and on Days 3 and 5, the cell number in the Se@SF/CPC group was significantly higher than those in the SF/CPC group (Fig. 6B). The results of cell number and viability detection *in vitro* demonstrated that the Se-modified bone cement had good biocompatibility. RT-PCR experiments showed that the transcription levels of *Gpx1* and mitochondrial respiratory chain-related complexes were significantly up-regulated in the Se@SF/CPC group compared with the SF/CPC group (Fig. 6C; Supplementary Fig. S5A). Treatment with the leaching solution of Se@SF/CPC significantly reduced the ROS level, while increasing the protein expression of GPx1 in OP-BMMSCs (Fig. 6D; Supplementary Fig. S5B), indicating the good antioxidant property of the Se-modified bone cement.

### Se-modified bone cement accelerated the repair of osteoporotic bone defects

**Figure 7.**
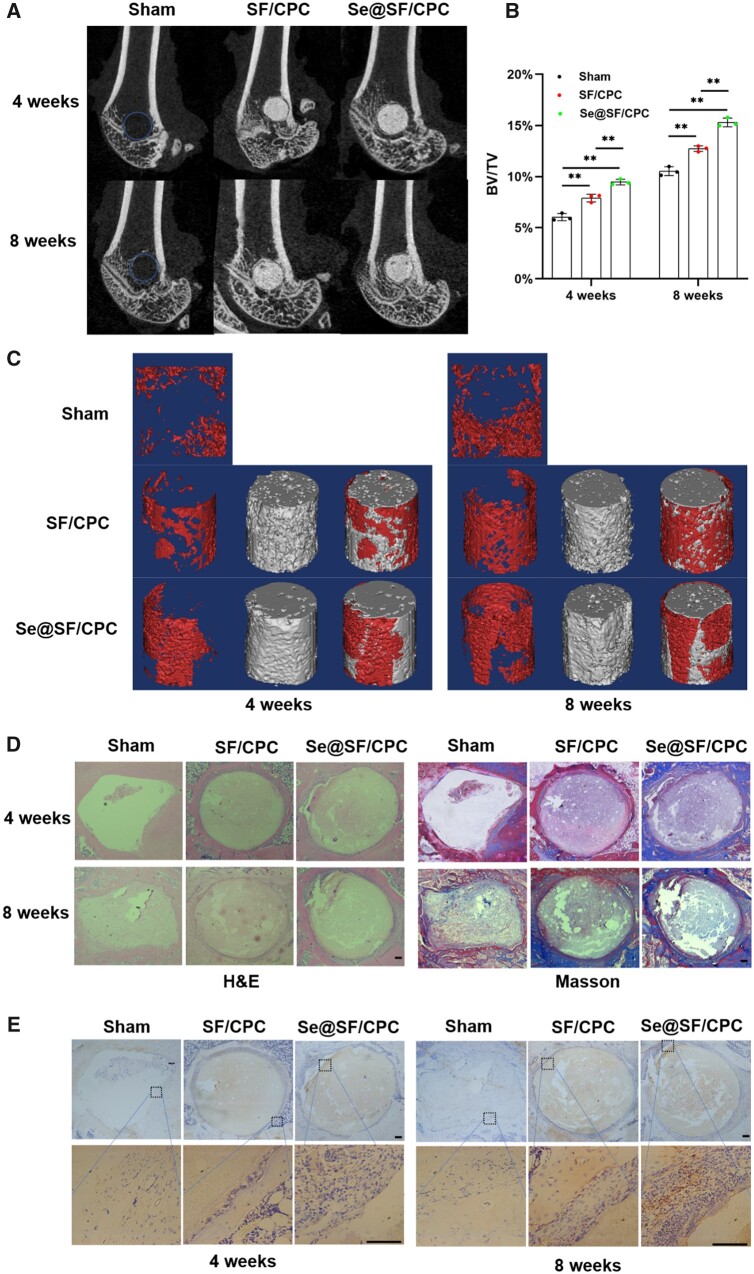
Se-Modified bone cement improved the osteoporotic bone defect repair in OVX rats. Defects of lateral femoral condyle in OVX rats were repaired using bone cement with or without Na_2_SeO_3_. (**A**, **C**) After 4 and 8 weeks of surgery, micro-CT analysis was used to evaluate the osteoporotic bone defect repair. The red areas represent the new bone, and the gray areas represent the remaining material. (**B**) Quantitative analysis of BV/TV at the time points of 4 and 8 weeks. *n* = 3. (**D**) Representative histological images of rat femurs using hematoxylin eosin (H&E), and masson staining. Scale bar = 100 μm. (**E**) Representative immunohistochemical staining of the expression of GPx1 around the bone defects. Scale bar = 100 μm. Values represent mean ± SEM. Statistically significant differences are indicated by * where *P *<* *0.05 or ** where *P *<* *0.01 between the indicated groups.

At Week 4, the new BV/TV in the SF/CPC group and the Se@SF/CPC group was 1.9% and 3.4% higher than that in the sham group, respectively. Furthermore, at Week 8, the new BV/TV in the SF/CPC group and the Se@SF/CPC group was 2.2% and 4.8% higher than that in the sham group, respectively (Fig. 7A–C). H&E and Masson staining indicated that at Week 4, there were many fibrous tissues and few collagen fibers around the bone defect in the sham group, while the new bone was separated by fibrous tissue in the two bone cement groups. At Week 8, many fibrous tissues were observed in the sham group, but the collagen fibers and new bone tissue increased gradually. Although fibrous tissues were still dominant around bone defects in the two bone cement groups, the number of new bone tissue around a bone defect in the Se@SF/CPC group was more than that in the SF/CPC group and the sham group (Fig. 7D). Immunohistochemical analysis demonstrated that the expression of GPx1 around bone defect was significantly increased in the Se@SF/CPC group compared with the SF/CPC group and the sham group (Fig. 7E). These results suggested that the Se-modified bone cement accelerated the repair of osteoporotic bone defects in OVX rats.

## Discussion

Several clinical and experimental studies have indicated that bone healing is dramatically delayed in osteoporotic patients [27, 28]. Consistent with previous studies, OVX was performed to establish osteoporotic rat model in this study, and we depicted that BMMSCs derived from OVX rats have a poor proliferation ability and an impaired osteogenic potential [29, 30]. Mitochondrial peroxide is one of the chief sources of ROS, and redox homeostasis in mitochondria of BMMSCs may take part in osteoporotic bone loss [31]. In this study, we demonstrated that the mitochondrial function of OP-BMMSCs was injured, accompanied by an imbalance of redox metabolism and decreased bone formation. ROS produced in mitochondria can be scavenged by antioxidant enzymes that maintain a dynamic balance under physiological conditions [32]. However, excessive level of ROS during OP development disturbed intracellular redox homeostasis. We also found that the mitochondrial respiratory chain in OP-BMMSCs was seriously impaired, which not only hindered ATP production, but also postponed bone healing in OVX rats. Tang *et al*. [33] demonstrated that improving specific antioxidant enzymes preserved bone microstructure and calcium homeostasis by reducing intracellular ROS level and maintaining mitochondrial energy metabolism. Therefore, restoring the OP-impaired mitochondrial function of BMMSCs holds promise to improve their osteogenic differentiation potential and accelerate osteoporotic bone defect repair.

Since Se plays a vital role in maintaining bone health, porous Se@SiO_2_ nanocomposite has been demonstrated to promote cell migration and the osteogenic differentiation of rat BMMSCs, resulting in acceleration of bone fracture healing [34, 35]. However, little was known about the direct therapeutic effects of Se on an osteoporotic bone defect. Na_2_SeO_3_ is a common Se supplement that is used in clinical practice. In this study, we determined that the optimum concentration of Na_2_SeO_3_ for OP-BMMSCs *in vitro* culture was 100 nM, because treatment with Na_2_SeO_3_ at higher concentrations revealed a toxic effect on cell viability [17]. Liu *et al.* [18] displayed that pretreatment with selenite protected BMMSCs from the adverse effects of H_2_O_2_ and promoted osteogenic differentiation, such as alkaline phosphatase activity, gene expression of type I collagen, and matrix mineralization. In addition, Se has been reported to play a key role in protection mechanisms against OS via the mitochondrial pathway. Supplementation with selenite effectively restored the antioxidant capacity of BMMSCs and reduced cell damage by enhancing the activity of antioxidant enzymes [17]. Therefore, we further delved into the effects of Na_2_SeO_3_ on the impaired mitochondrial function of OP-BMMSCs afterward. Our results suggested that Na_2_SeO_3_ can significantly attenuate intracellular ROS and mitochondrial superoxide, and increase MMP levels and ATP production. In particular, treatment with Na_2_SeO_3_, successfully recovered the activity of mitochondrial respiratory chain in OP-BMMSCs.

GPx1 is an important Se-dependent antioxidant enzyme that prevents harmful accumulation of ROS, whereas inhibition of *Gpx1* leads to reduced mitochondrial ATP production and imbalance of mitochondrial redox homeostasis [36, 37]. A previous study from our laboratory showed that the mRNA and protein expression of GPx1 were significantly lower in OP-BMMSCs than those in normal BMMSCs, suggesting that the down-regulation of GPx1 was possibly related to the pathogenesis of OP [38]. Meanwhile, we found that the expression and activity of GPx1 gradually increased accompanied by the osteogenic differentiation of BMMSCs [39]. These results suggested that GPx1 not only took part in the pathogenesis of OP but was also involved in the bone formation and healing process. In this study, we demonstrated for the first time to our knowledge that supplementation with Na_2_SeO_3_ significantly up-regulated the expression and enzyme activity of GPx1 in OP-BMMSCs that was responsible for the restitution of mitochondrial function and improvement of the osteogenic potential. To further explore whether GPx1 was involved in Se-regulated mitochondrial antioxidant functions, we used siRNA to silence *Gpx1* expression. After silencing *Gpx1* expression, the protective effects of Na_2_SeO_3_ were completely counteracted, confirming that Na_2_SeO_3_ restored the osteogenic differentiation of OP-BMMSCs through GPx1-mediated mitochondrial antioxidant functions. In addition, GPx1 has been reported to be involved in bone remodeling by increasing osteoclast activity and bone resorption. Handy *et al.* [40] demonstrated that *Gpx1* overexpression in permanently transfected or adenovirus-treated cells resulted in overall mitochondrial dysfunction with a decrease in mitochondrial potential and ATP production. Therefore, our future studies will investigate the roles of selenoprotein GPx1 in regulating osteoclast maturation and bone resorption.

In the condition of OP, the strategy to promote bone repair remains a challenge. In a recent study, Tao *et al*. [41] reported that lithium- and aspirin-modified CPC is a scheme for rapid repair of osteoporotic bone defects, and these effects may be achieved by inhibiting local inflammation and through the bone morphogenetic protein-2/small mothers against decapentaplegic homologs-1 (BMP-2/Smad1) and osteoprotegerin/receptor activator of nuclear factor (NF) kappa-B ligand (OPG/RANKL) signaling pathways. van Houdt *et al.* [42] prepared a synthetic bone substitute material using CPC and polylactic-co-glycolic acid (PLGA) to stimulate bone formation in osteoporotic bone defects by loading with alendronate. Therefore, considering the beneficial effects of Na_2_SeO_3_ on the osteogenic differentiation of OP-BMMSCs, for the first time, we prepared a modified bone cement by adding Na_2_SeO_3_ into the liquid phase of SF and enhancing its biological activity. Considering that the optimal addition concentration of Na_2_SeO_3_ in this modified bone cement has not been clearly studied, we used the leach solution to investigate the Se-modified bone cement on the cell viability. We found that Na_2_SeO_3_ released from the bone cement facilitated OP-BMMSC growth; however, the direct effect of Se-modified bone cement with different concentrations of Na_2_SeO_3_ on bone cells will be investigated in future studies. As for the characteristics of modified bone cement, relevant experiments have shown that supplementation with Na_2_SeO_3_ did not significantly affect the microstructure, injectability, compressive strength and setting time of SF/CPC. In addition, CPC is a good system for Se release that can load a variety of drugs and achieve a sustained release effect [43]. However, α-TCP degrades slowly, and the degradation time is much longer than the complete drug release time, and the drug release mechanism is similar to the non-degradable carrier and belongs to the diffusion regulation type [44]. Precisely regulating the degradation rate of bone cement and the effective, stable and slow release of the drug will be the focus of future researches on drug-loaded bone cement. *In vivo* animal experiments displayed that Se-modified SF/CPC effectively promoted new bone formation around the bone defects, as evidenced by both micro-CT analysis and histology staining results. Immunohistochemical analysis demonstrated that the expression of GPx1 around bone defect was significantly increased in the Se@SF/CPC group compared with other groups. The above results confirmed that through the locally release of Se, Se-modified bone cement regulated mitochondrial antioxidant function mediated by selenoprotein GPx1, thereby promoting osteogenic differentiation of OP-BMMSCs and accelerating the repair of osteoporotic bone defects.

Traditionally, many studies have focused on the effects of inorganic Se; however, in recent years, organic Se (such as selenomethionine) and Se nanoparticles (SeNPs) have been reported to induce many unique biological activities. Li *et al*. [45] revealed that selenomethionine attenuated H_2_O_2_-induced dysfunction in BMMSCs through an antioxidant effect, suggesting that it is a promising candidate for reducing OS. Compared with ordinary Se, SeNPs are highly efficient molecular compounds with higher antioxidant activity and lower toxicity. Fatima *et al*. [46] confirmed that low concentrations of SeNPs enhanced the cell viability and osteogenic potential of human mesenchymal stem cells and increased c-Jun N-terminal kinase (JNK) and Forkhead box O3 (FOXO3) expression, suggesting that SeNPs might enhance osteogenesis via activation of the JNK/FOXO3 pathway. In addition, the protective effects of Se on bone health may involve other selenoproteins that affect the osteogenic potential of BMMSCs. Zhang *et al*. [47] reported that thioredoxin reductase 1 (TrxR1), an important selenoprotein, is expressed in the early differentiated osteoblasts, highlighting a part of the mechanism whereby Se promotes bone formation. Selenoprotein W has also been reported to regulate osteoclast differentiation by promoting the nuclear translocation of receptor activator of NF-κΒ, while specific deficiency of selenoprotein W in mice exhibited OP-related bone mass loss [48]. Moreover, a new lncRNA Bmcob was demonstrated to mediate the osteogenic differentiation and regulate the antioxidant functions of BMMSCs by targeting the mRNAs of a series of selenoproteins [49]. These findings reveal that selenoproteins play an important role in regulating osteogenic differentiation of BMMSCs and have gradually become a potential target for treating osteoporotic bone defects.

## Conclusions

We demonstrated that OP-BMMSCs showed impaired mitochondrial functions and reduced osteogenic differentiation potential accompanied by an increase in intracellular OS. *In vitro* supplementation with Se successfully restored the osteogenic differentiation of OP-BMMSCs through reconstitution of mitochondrial antioxidant functions. Further molecular experiments revealed that GPx1 played a critical role in Se-mediated restoration of mitochondrial functions in OP-BMMSCs since inhibiting *Gpx1* by siRNA abrogated the protective effects against OS. Based on the beneficial effects of Se on bone formation, an Se-modified SF/CPC scaffold was developed and effectively accelerated the repair of an osteoporotic bone defect. Incorporating Se and bone cement represents a novel strategy for treating bone fractures or bone defects in patients with OP.

## Supplementary Material

rbad011_Supplementary_Data

## Data Availability

The original contributions presented in the study are included in the article/supplementary material, further inquiries can be directed to the corresponding author.
