# Supplementary material for: Selenium-modified bone cement promotes osteoporotic bone defect repair in ovariectomized rats by restoring GPx1-mediated mitochondrial antioxidant functions
Source: Regen Biomater. 2023 Feb 14;10:rbad011. doi: 10.1093/rb/rbad011 (PMC9960915; doi:10.1093/rb/rbad011)
Supplement: rbad011_Supplementary_Data [file rbad011_supplementary_data.zip › Supplementary Table.doc]

| **Supplementary table 1. Primer sequences of each gene** | | |
| --- | --- | --- |
| Gene | Sense (5'-3') | Antisense (5'-3') |
| *Gapdh* | AGACAGCCGCATCTTCTTGT | TACTCAGCACCAGCATCACC |
| *Runx2* | CCAACTTCCTGTGCTCCGTG | GTGAAACTCTTGCCTCGTCCG |
| *Sp7* | CCCAACTGTCAGGAGCTAGAG | GATGTGGCGGCTGTGAAT |
| *Bglap* | GACCCTCTCTCTGCTCACTCT | GACCTTACTGCCCTCCTGCTTG |
| *Gpx1* | TTGAGAATGTCGCGTCC | CAAGCCCAGATACCAGGA |
| *Cox4* | GTGCTGATCTGGGAGAAGAGCTA | GGTTGACCTTGATGTCCAGCAT |
| *Nd4* | CCCTACCCTCAACATGATCC | GGAGCTTCTACGTGGGCTTT |
| *Sdha* | TGGACCTTGTCGTCTTTGG | TTTGCCTTAATCGGAGGAAC |
| *Atp5a* | TCTCCATGCCTCTAACACTCG | CCAGGTCAACAGACGTGTCAG |
