# Supplementary material for: Selenium-modified bone cement promotes osteoporotic bone defect repair in ovariectomized rats by restoring GPx1-mediated mitochondrial antioxidant functions
Source: Regen Biomater. 2023 Feb 14;10:rbad011. doi: 10.1093/rb/rbad011 (PMC9960915; doi:10.1093/rb/rbad011)
Supplement: rbad011_Supplementary_Data [file rbad011_supplementary_data.zip › Supplementary Figures_RB-2022-234.R1.doc]

**Supplementary Figures**

**Supplementary Fig. 1. BMMSCs derived from OVX rats (OP-BMMSCs) exhibited decreased proliferative potential, GPx1 protein expression, and impaired mitochondrial functions.** (A) The isolated cells from rats were identified as BMMSCs by flow cytometry. n=3. (B) Cell proliferation of N-BMMSCs and OP-BMMSCs was compared on days 1, 3, 5, and 7 using CCK-8 assay. n=8. (C) The protein levels of GPx1 were determined using Western blot. n=3. (D) Mitochondrial membrane potential (MMP) level was determined by flow cytometry. n=3. (E) The protein levels of ND4, SDHA, COX4, and ATP5A in N-BMMSCs and OP-BMMSCs were determined using Western blot. n=3. Values represent mean ± S.E.M. Statistically significant differences are indicated by * where *P* < 0.05 or ** where *P* < 0.01 between the indicated groups.

**Supplementary Fig. 2. *In vitro* treatment with Na_2_SeO_3_ attenuated GPx1 protein expression, and impaired mitochondrial functions of OP-BMMSCs.** (A) The protein level of GPx1 in Na_2_SeO_3_-treated OP-BMMSCs was determined using Western blot. n=3. (B) The protein levels of ND4, SDHA, COX4, and ATP5A in Na_2_SeO_3_-treated OP-BMMSCs were determined using Western blot. n=3. Values represent mean ± S.E.M. Statistically significant differences are indicated by * where *P* < 0.05 or ** where *P* < 0.01 between the indicated groups.

**Supplementary Fig. 3. Inhibition of *Gpx1* by siRNA abrogated the protective effect of Na_2_SeO_3_ on the proliferative potential and mitochondrial antioxidant functions of OP-BMMSCs.** (A) The protein level of GPx1 in siRNA-transfected OP-BMMSCs were evaluated by Western blot. n=3. (B) Mitochondrial membrane potential (MMP) level in siRNA-transfected OP-BMMSCs was determined by flow cytometry. n=3. (C) The protein levels of ND4, SDHA, COX4, and ATP5A in siRNA-transfected OP-BMMSCs were evaluated by Western blot. n=3. (D) Cell proliferation was examined on days 1, 3, 5, and 7 using CCK-8 assay. n=8. Values represent mean ± S.E.M. Statistically significant differences are indicated by * where *P* < 0.05 or ** where *P* < 0.01 between the indicated groups.

**Supplementary Fig. 4. Characterization of modified bone cement and establishment of animal model.** (A) The optimum concentration of Na_2_SeO_3_ in modified bone cement was determined by CCK-8 assay. n=6. (B) The phase composition of modified bone cement was detected using X-ray powder diffraction (XRD). (C) The energy dispersive X-ray spectroscopy (EDS) elemental analysis maps of all elements, including oxygen (O), calcium (Ca), carbon (C), phosphorus (P), nitrogen (N), and selenium (Se). (D) Bilateral ovariectomies (OVX) were performed in female rats, and a 3 mm diameter defect was drilled from the lateral condyle of the distal femur in bilateral OVX rats. Values represent mean ± S.E.M. Statistically significant differences are indicated by * where *P* < 0.05 or ** where *P* < 0.01 between the indicated groups.

**Supplementary Fig. 5. Determination of** **antioxidant properties of the leaching solution of modified bone cement.** OP-BMMSCs were cultured using the leached solution of SF/CPC and Se@SF/CPC, while cell culture medium without served as the CTRL group. (A) The *Nd4, Sdha, Cox4,* and *Atp5a* genes expressions of OP-BMMSCs were quantified with real-time RT-PCR. n=4. (B) The reactive oxygen species (ROS) levels of OP-BMMSCs were determined by immunofluorescence assay. Scale bar: 200 μm. Values represent mean ± S.E.M. Statistically significant differences are indicated by * where *P* < 0.05 or ** where *P* < 0.01 between the indicated groups.
